# Cerebral arterial mapping in the equine brain: a detailed anatomical study

**DOI:** 10.3389/fvets.2026.1828754

**Published:** 2026-06-02

**Authors:** Ahmad Al Aiyan, Sara Abu Hayah, Abdulrahman Fahad Alnahdi, Arwa Alshamsi, Hessa Alshebli, Sara Aleissaee, Rinsha Balan

**Affiliations:** Department of Veterinary Medicine, College of Agriculture and Veterinary Medicine, United Arab Emirates University, Al Ain, United Arab Emirates

**Keywords:** cerebral arteries, circle of Willis, horse brain, neuroanatomy, vascular anatomy

## Abstract

**Introduction:**

Detailed knowledge of the cerebral arterial system in equine brain is required for advancing neuroanatomical understanding and supporting the interpretation of neurological disorders. The equine cerebrum, characterized by extensive cortical organization and functional specialization, requires a continuous arterial blood supply; however, detailed information on its arterial anatomy remains limited. This study aimed to describe the arterial supply to the equine cerebrum, including the origin, branching patterns, and distribution of the major cerebral arteries.

**Methods:**

Ten adult horse heads were examined following formaldehyde fixation and colored latex injection through the common carotid arteries. Dissections were performed, and the arteries were documented using multi-angled photographic analysis.

**Results:**

A complete and symmetrical circle of Willis was formed by internal carotid branches connecting the carotid and basilar systems. The rostral cerebral artery supplied the rostromedial cortex and corpus callosum; the middle cerebral artery supplied the lateral and dorsolateral cortical surfaces; and the caudal cerebral artery supplied the occipital and caudal temporal lobes. Minor anatomical variations were observed, including interhemispheric anastomoses in 60% of specimens; however, the overall arrangement remained consistent across the examined specimens (*n* = 10).

**Discussion:**

These findings provide a detailed anatomical characterization of cerebral arterial branching patterns in the equine brain and serve as an anatomical reference that may support the interpretation of cerebral imaging and inform future comparative and clinical investigations.

## 1 Introduction

The brain is one of the most metabolically active organs in mammals and requires a constant, well-regulated blood supply to maintain its function. Even brief interruptions in cerebral perfusion can cause irreversible neuronal damage, highlighting the essential role of the cerebral arterial system in maintaining a continuous oxygen supply (1–4). In mammals, the cerebral circulation is adapted to meet metabolic needs and accommodate variations in body size, head position, and other environmental factors (5–7). Among domestic species, equines have one of the most developed and largest brains relative to body size, characterized by extensive cortical gyrification and a high degree of hemispheric specialization (8–10). The primary blood supply to the equine brain comes from the internal carotid (ICA) and vertebral arteries (VA), which together form the cerebral arterial circle (circle of Willis) at the base of the brain (2). Within this arterial circle, three principal cerebral arteries, the rostral cerebral artery (RCA), middle cerebral artery (MCA), and caudal cerebral artery (CCeA), originate and supply the respective cortical and subcortical regions within each cerebral hemisphere (3, 11). Comparative anatomical studies have indicated that, unlike ruminants and camels, the ICA in equines remains patent and functional throughout life, providing a direct contribution to the cerebral arterial circle (2, 12, 13). In ruminants, the extracranial segment of the internal carotid artery undergoes postnatal regression and is replaced by the rete mirabile carotidis, which becomes the principal source of cerebral blood supply (5, 6, 14). Among camelids, both the ICA and rete mirabile contribute to the formation of the circle of Willis, reflecting a mixed pattern of cerebral vascular supply (15–18). In contrast, equines, carnivores, and primates exhibit a more primitive configuration characterized by direct internal carotid contribution and a completely symmetrical circle of Willis (2). Although previous studies have described the general organization of the equine cerebral arterial system (2, 12), detailed mapping of intracerebral branching patterns, their cortical distribution, and associated variability remains comparatively limited. Investigations in other species, such as cattle, goats, camels, and deer, have revealed marked variability in the origin and course of cerebral arteries, particularly within the rostral and middle cerebral systems (19–21). However, comparable detailed analyses of the equine cerebrum remain scarce. The lack of systematic mapping and quantitative documentation continues to constrain anatomical insights and clinical interpretations, particularly in the context of neuroimaging, cerebrovascular disorders, and surgical interventions such as those associated with guttural pouch mycosis, or arterial occlusion procedures, where arterial involvement may lead to severe hemorrhage or ischemic complications (12, 22, 23). Accordingly, this study aimed to provide an in-depth anatomical characterization of the cerebral arterial supply to the equine cerebrum, emphasizing the origin, branching pattern, and distribution territories of the principal cerebral arteries. Collectively, these findings establish a detailed framework of the equine cerebral arterial architecture, addressing the limited availability of detailed descriptions of intracerebral arterial branching patterns and strengthening its relevance for anatomical interpretation and providing a basis for future investigations in equine neurology and neurosurgery.

## 2 Materials and methods

### 2.1 Specimen collection

The heads of 10 adult horses, approximately 4–12 years old, were donated by veterinary teaching clinics following humane euthanasia for conditions unrelated to neurological disorders. None of the animals had a history of central nervous system disease or cranial trauma. All procedures were carried out according to the biosafety guidelines of the Animal Research Ethics Committee and conformed to the institutional regulations governing the scientific use of animal tissues.

### 2.2 Fixation and vascular injection

The two common carotid arteries were immediately cannulated, and the arterial system was flushed with physiological saline to remove residual blood, followed by perfusion with 10% formaldehyde in neutral solution to fix the cerebral tissue. The heads were preserved in the same fixative for at least 2 weeks.

To visualize the intracranial arterial system, colored latex was injected through both common carotid arteries using 60-mL syringes using a controlled manual injection technique. The injection was performed at a steady low pressure to avoid vascular rupture. The volume of latex injected per specimen ranged from approximately 250–400 mL, depending on vascular resistance and specimen size.

Adequate perfusion was determined based on several criteria, including the appearance of latex within peripheral tissues such as the nasal mucosa and oral structures, as well as increased resistance to further injection, suggesting complete vascular filling. Successful perfusion was further confirmed during dissection by the continuous presence of latex within the circle of Willis and its principal branches.

The preparation and handling of the injection medium were performed as previously described (24–26). The specimens were maintained at room temperature for 24–48 h after injection to allow complete polymerization of the latex.

### 2.3 Dissection and documentation

The dorsal calvarium was carefully removed using a bone saw, and the brain was carefully extracted. Dissections were performed systematically to expose and trace the cerebral arterial supply. Both hemispheres were examined to record bilateral patterns and variations. Each stage of dissection was photographically documented using images taken from the ventral, dorsal, medial, and lateral perspectives. Observations were recorded bilaterally to evaluate the frequency and nature of any asymmetrical features.

## 3 Results

For clarity, arterial branches are described according to their regional distribution and functional grouping, and detailed anatomical variations are summarized in Table 1, and abbreviations used throughout the manuscript are listed in Table 2.

**Table 1:** Frequency and distribution of anatomical variations in the cerebral arterial system of the horse (*n* = 10).

Artery	Observed variation	Percentage (%)
Rostral trunk (MCA branches)	Common orbitofrontal trunk (OFraA + IF) instead of separate origins	3/10 (30%)
Caudal trunk (MCA branches)	Presence of the accessory middle parietal artery	4/10 (40%)
Rostral cerebral artery (RCA)	Additional interhemispheric anastomotic branch between left and right RCA	6/10 (60%)
RCA (medial branches)	Common trunk for orbitofrontal (OFraA) and frontopolar (FP) arteries	6/10 (60%)
Rostral callosal artery (RCaA)	Origin from left RCA	5/10 (50%)
Rostral callosal artery (RCaA)	Origin from right RCA	3/10 (30%)
Rostral callosal artery (RCaA)	Bilateral independent origins	2/10 (20%)
Caudal frontal artery (CF)	Origin directly from RCA	6/10 (60%)
Caudal frontal artery (CF)	Shared trunk with rostral parietal artery (RP)	4/10 (40%)
Rostral parietal artery (RP)	Origin from CF–RP common trunk	4/10 (40%)
Middle callosal artery (MCaA)	Origin directly from RCA before parietal branches	4/10 (40%)
Caudal cerebral artery (CCeA branches)	Medial rostral temporal artery (mRT) arises directly from CCeA	5/10 (50%)
Caudal cerebral artery (CCeA branches)	mRT and mCT arise from common trunk	5/10 (50%)

**Table 2:** Abbreviations used for cerebral arteries in the equine brain.

Full name of the artery	Abbreviation	Full name of the artery	Abbreviation
Basilar artery	BA	Superior frontal artery	SF
Caudal communicating artery	CCuA	Rostral frontal artery	RF
Internal carotid artery	ICA	Caudal frontal artery	CF
Rostral cerebral artery	RCA	Rostral parietal artery	RP
Middle cerebral artery	MCA	Middle parietal artery	MP
Caudal cerebral artery	CCeA	Caudal parietal artery	CP
Rostral communicating artery	RCoA	Rostral temporal artery	RT
Rostral choroidal artery	RChA	Caudal temporal artery	CT
Rostral olfactory artery	ROlA	Medial rostral temporal artery	mRT
Rostral callosal artery	RCaA	Medial caudal temporal artery	mCT
Middle callosal artery	MCaA	Medial occipital artery	mOcA
Caudal callosal artery	CCaA	Lateral occipital artery	lOcA
Orbital artery	OrbA	Inferior frontal artery	IF
Orbitofrontal artery	OFraA	Frontopolar artery	FP
Middle frontal artery	MF		

### 3.1 Circle of Willis

**Figure 1:**
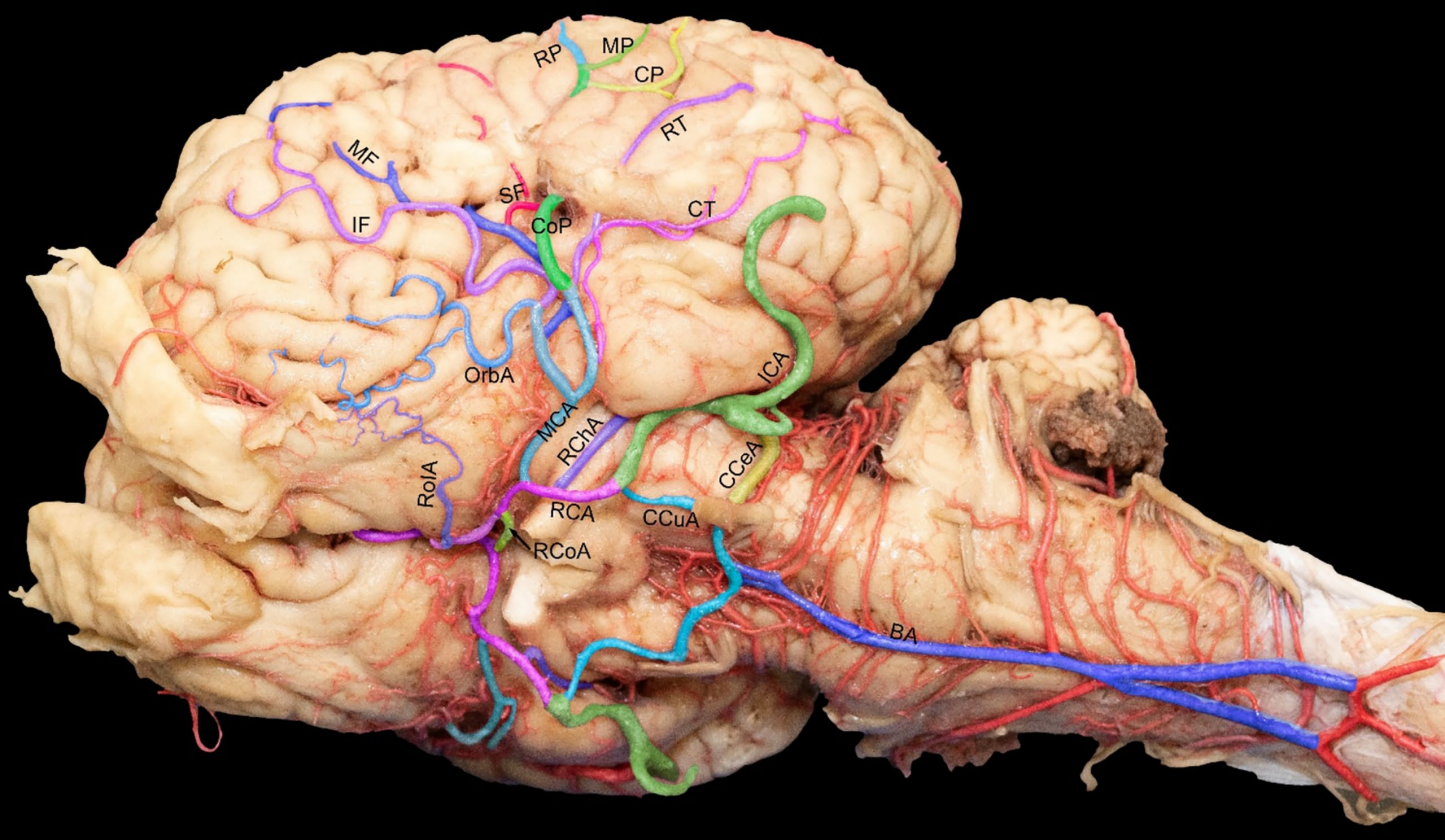
Lateroventral view of the equine brain showing the circle of Willis and the main arterial branches. BA, Basilar artery; ICA, internal carotid artery; CCuA, caudal communicating artery; RCA, rostral cerebral artery; MCA, middle cerebral artery; CCeA, caudal cerebral artery; RChA, rostral choroidal artery; RCoA, rostral communicating artery; ROlA, rostral olfactory artery; OrbA, orbital artery; IF, inferior frontal artery; MF, middle frontal artery; SF, superior frontal artery; RP, rostral parietal artery; MP, middle parietal artery; CP, caudal parietal artery; RT, rostral temporal artery; CT, caudal temporal artery.

All 10 specimens examined showed a fully developed symmetrical circle of Willis located on the ventral brain surface. The arterial circle displayed a polygonal shape situated at the junction of the cerebral hemispheres, brainstem, and optic chiasm. The circle was mainly formed by the terminal branches of the ICA, which contributed to the rostral and lateral components, and the caudal communicating arteries (CCuA), which extended caudally to connect with the basilar artery (BA). In all examined specimens (*n* = 10), the circle appeared compact, continuous, and similar in size, with no observable interruptions or irregularities. The ICAs entered the cranial cavity through the foramen lacerum and ascended obliquely in a rostromedial direction toward the base of the brain. Within the cranial cavity, each artery displayed a smooth curvature before dividing into two major branches: RCA and CCuA (Figure 1). The RCA projected rostrally to form the anterolateral segment of the arterial circle, whereas the CCuA ran caudally to anastomose with the BA, thereby completing the caudal part of the circle of Willis (Figure 1).

In all examined specimens (*n* = 10), the circle of Willis was completely closed at both the rostral and caudal ends. Rostral closure consisted of a short communicating segment uniting the paired RCA, whereas caudal closure resulted from the junction of the CCuA with the BA (Figure 1). Together, these connections created a continuous anastomotic ring encircling the pituitary gland. No duplication, interruption, or asymmetry of the arterial circle was detected within the examined sample (*n* = 10).

### 3.2 Rostral cerebral artery

The RCA was the terminal continuation of the ICA. Overall, the branching pattern of the RCA followed a consistent organizational scheme, with lateral cortical branches arising primarily from the middle cerebral artery and medial branches supplying the corpus callosum and adjacent structures, although variations in branching patterns were observed. It extended rostrally along the ventral surface of the cerebrum. Shortly after its origin, it gave rise to two major branches, the rostral choroidal artery (RChA) and the middle cerebral artery (MCA) (Figure 1). The RChA was consistently identified as the first branch emerging from the RCA. It coursed dorsolaterally, passing between the midbrain and piriform lobe, and continued toward the choroid plexus of the third and lateral ventricles, which represented its principal distribution area (Figure 1).

The MCA originated as the second main branch of the RCA. It ran laterally and dorsally toward the Sylvian fissure, where it bifurcated into two trunks: a rostral trunk and a caudal trunk (Figure 1).

**Figure 2:**
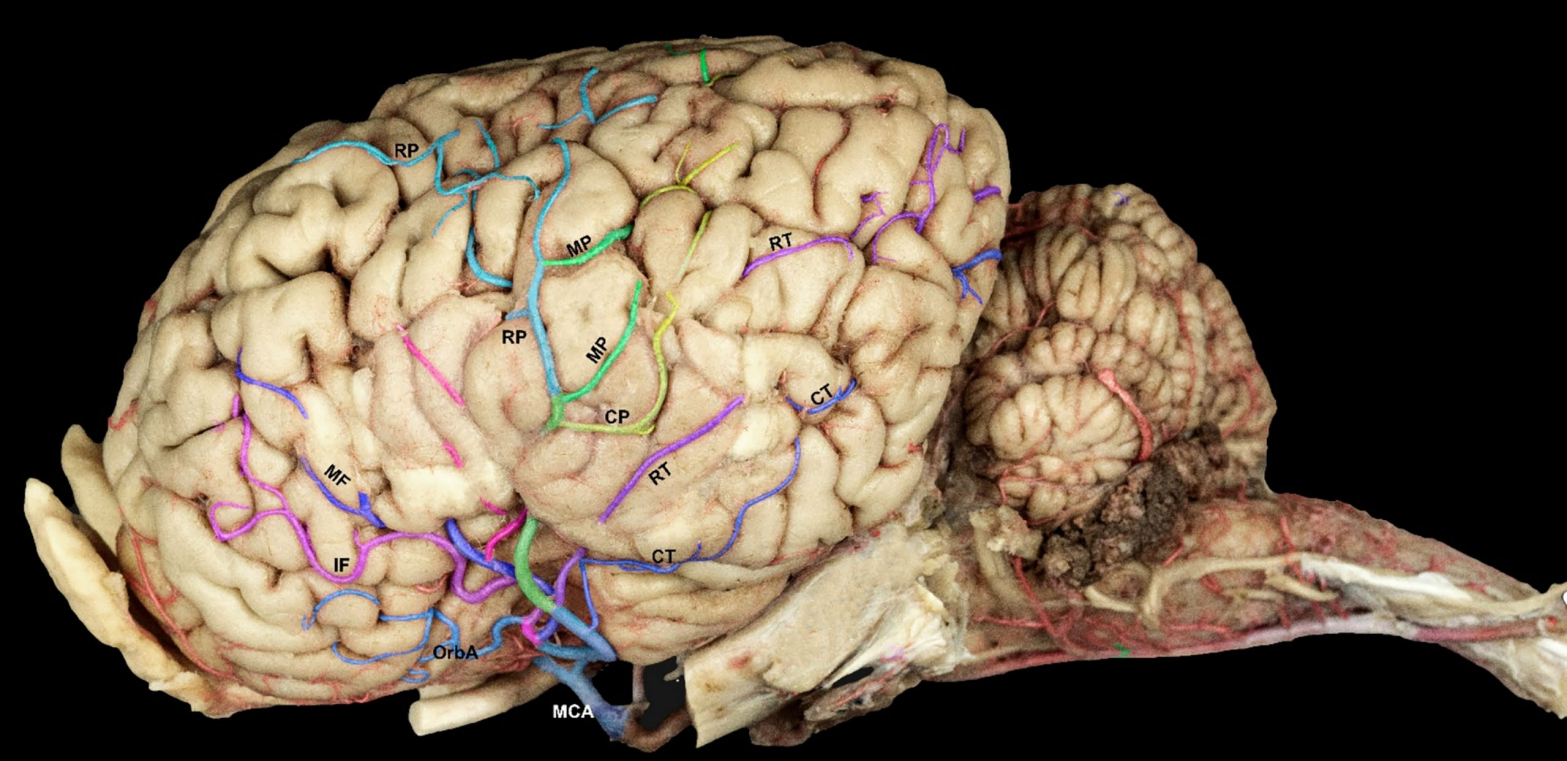
Left lateral view of the equine brain showing the branching pattern of the middle cerebral artery and its cortical branches on the lateral surface of the hemisphere. MCA, middle cerebral artery; OrbA, orbital artery; IF, inferior frontal artery; MF, middle frontal artery; SF, superior frontal artery; RP, rostral parietal artery; MP, middle parietal artery; CP, caudal parietal artery; RT, rostral temporal artery; CT, caudal temporal artery.

The rostral trunk of the MCA gave rise to several frontal branches, including the orbital (OrbA), inferior frontal (IF), middle frontal (MF), and superior frontal (SF) arteries, which collectively supplied the lateral and dorsal regions of the frontal lobe (Figures 1, 2). These arteries followed characteristic courses along the diagonal and Sylvian sulci, providing vascular supply to the orbital, ventrolateral, and dorsorostral cortical regions. Variations in their origin were observed, including the presence of a common orbitofrontal trunk in some specimens, as summarized in Table 1.

The caudal trunk of the MCA extended laterally toward the Sylvian fissure and gave rise to branches supplying the temporal, parietal, and occipital lobes of the cerebrum (Figures 1, 2). Temporal branches, including the caudal temporal (CT) and rostral temporal (RT) arteries, supplied the temporal cortex and extended toward adjacent occipital regions (Figures 1, 2).

Parietal branches, comprising the rostral (RP), middle (MP), and caudal (CP) parietal arteries, supplied the dorsal and medial aspects of the parietal lobe (Figures 1, 2). These vessels followed distinct dorsally oriented courses across the cortical surface. Variations in their origin and arrangement were observed, including shared trunks and accessory branches, as detailed in Table 1.

Following the origin of the MCA, the RCA continued rostrally toward the longitudinal fissure. Before entering the fissure, it gave rise to the rostral communicating artery (RCoA), which connected the right and left RCAs and completed the rostral portion of the circle of Willis (Figure 1). Additional rostral olfactory branches (ROlAs) extended toward the olfactory tracts and bulbs, supplying the rostral olfactory regions.

Within the longitudinal fissure, the RCA followed a relatively consistent course between the cerebral hemispheres. An additional anastomotic branch between the left and right RCAs was identified in 60% of the specimens, as summarized in Table 1.

**Figure 3:**
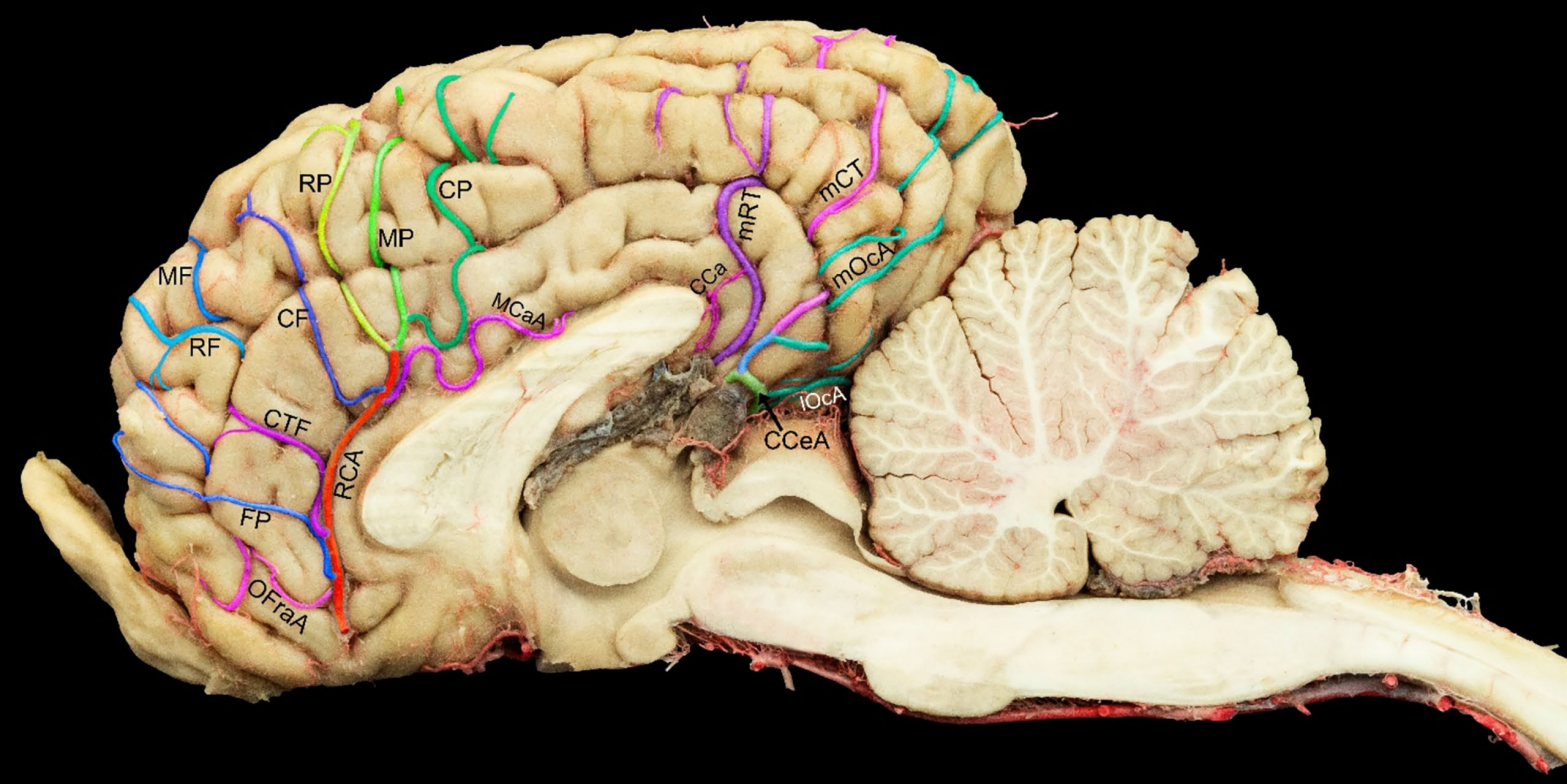
Medial view of the right cerebral hemisphere showing the arteries on the medial surface. RCA, rostral cerebral artery; OFraA, orbitofrontal artery; FP, frontopolar artery; RF, rostral frontal artery; MF, middle frontal artery; CF, caudal frontal artery; RP, rostral parietal artery; MP, middle parietal artery; CP, caudal parietal artery; MCaA, middle callosal artery; CCeA, caudal cerebral artery; CCaA, caudal callosal artery; mRT, medial rostral temporal artery; mCT, medial caudal temporal artery; mOcA, medial occipital artery; lOcA, lateral occipital artery.

**Figure 4:**
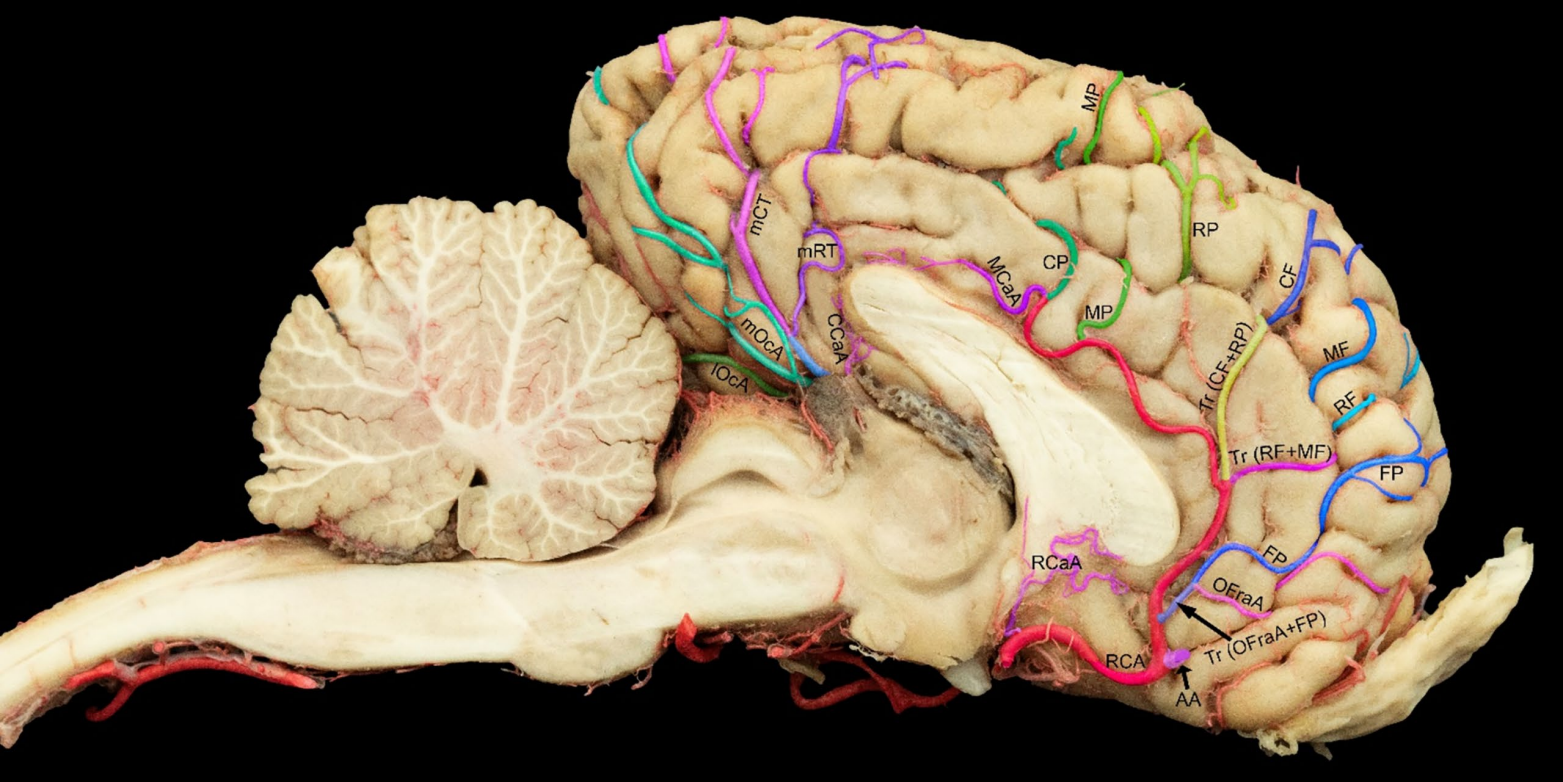
Medial view of the left cerebral hemisphere showing the arteries on the medial surface. RCA, rostral cerebral artery; AA, anastomosing artery; OFraA, orbitofrontal artery; FP, frontopolar artery; RF, rostral frontal artery; MF, middle frontal artery; CF, caudal frontal artery; RP, rostral parietal artery; MP, middle parietal artery; CP, caudal parietal artery; RCaA, rostral callosal artery; MCaA, middle callosal artery; CCaA, caudal callosal artery; mRT, medial rostral temporal artery; mCT, medial caudal temporal artery; mOcA, medial occipital artery; lOcA, lateral occipital artery; Tr (OFraA+FP), common trunk of the orbitofrontal and frontopolar arteries; Tr (RF+MF), common trunk of the rostral and middle frontal arteries; Tr (CF+RP), common trunk of the caudal frontal and rostral parietal arteries.

Medial branches of the RCA included the orbitofrontal (OFraA) and frontopolar (FP) arteries, which supplied the orbitofrontal cortex and rostral medial regions of the cerebrum (Figures 3, 4). The rostral callosal artery (RCaA) also exhibited variability in its origin, arising from either the left or right RCA or bilaterally, and supplied the ventrorostral portion of the corpus callosum.

Additional frontal and parietal branches, including the rostral frontal (RF), middle frontal (MF), caudal frontal (CF), and parietal arteries (RP, MP, CP), supplied the medial and dorsal cortical surfaces. These vessels followed characteristic courses within the callosal and cingulate sulci. Variations in their origin were observed, including shared trunks between CF and RP, as detailed in Table 1.

The middle callosal artery (MCaA) arose either directly from the RCA or as a continuation following the parietal branches, supplying the dorsal surface of the corpus callosum. Together with the RCaA and caudal callosal artery (CCaA), these vessels formed the principal arterial supply to the corpus callosum (Table 1).

The caudal cerebral artery (CCeA), originating from the caudal communicating artery, supplied the caudal regions of the cerebral hemisphere. Its branches included the medial rostral temporal (mRT), medial caudal temporal (mCT), and occipital arteries, which supplied the temporal and occipital lobes (Figures 3, 4). Variations in the origin of these branches were observed, including shared trunks between mRT and mCT or between mCT and mOcA, as summarized in Table 1.

The occipital region was supplied by the medial and lateral occipital arteries (mOcA and lOcA), which provided consistent vascularization of the occipital cortex across the examined specimens.

Overall, the cerebral arterial configuration demonstrated a largely consistent organizational pattern across the examined specimens (*n* = 10), with variability primarily affecting the branching patterns of selected cortical arteries. The most notable findings included the presence of an additional anastomotic connection between the rostral cerebral arteries (60%), a shared orbitofrontal trunk (30%), and the presence of an accessory middle parietal artery (40%). These variations did not alter the overall arrangement of the major cerebral vessels but reflect a degree of intraspecific variability. A summary of these findings is provided in Table 1.

## 4 Discussion

This study provides a detailed anatomical description of the cerebral arterial supply in equines. The following discussion interprets these findings in relation to existing literature and their potential anatomical and clinical relevance. In all examined specimens (*n* = 10), the circle of Willis appeared complete and symmetrical, consistent with previously reported patterns.

The present study provides several novel anatomical observations, including the frequency and pattern of interhemispheric anastomotic connections between the rostral cerebral arteries, the variability in the origin and arrangement of callosal arteries, and the presence of shared cortical arterial trunks in specific regions. In addition, this study offers a structured quantitative assessment of these variations, which has been limited in previous equine studies.

Although the present study is descriptive, these anatomical patterns may be relevant in clinical conditions involving altered cerebral perfusion or vascular compromise, where collateral circulation plays a role; however, further clinical studies are required to confirm these implications.

The ICA served as the principal source of cerebral blood supply, giving rise to well-defined rostral and caudal communicating branches that contribute to both the rostral and caudal ends of the circle. The MCA, RCA, and CCeA displayed largely consistent branching patterns across the examined specimens, with minor variations in cortical distribution patterns, particularly in the frontal, parietal, and temporal branches.

### 4.1 Internal carotid artery

In equines, the ICA remains the dominant arterial structure and the primary source of blood to the circle of Willis, supplying the majority of the forebrain structures. The division of the ICA into the RCA and CCuA aligns with the pattern documented in previous equine and donkey studies (2, 12, 13). The caudal communicating arteries provided an effective link between the carotid and basilar systems; however, no evidence of reverse flow from the basilar artery was identified based on the observed arterial configuration. Specifically, the direct continuity between the internal carotid system and the cerebral arterial circle, combined with the orientation of the caudal communicating arteries toward the basilar artery, suggests a predominantly rostral direction of blood flow, as described in previous studies (2, 3).

### 4.2 Circle of Willis

The complete and symmetrical circle of Willis is consistent with previous findings in equines (2, 11) and supports the view that the equine cerebral arterial circle shares key structural features with that observed in other mammals, particularly in maintaining a complete and symmetrical configuration. The polygonal ring was consistently formed rostrally and laterally by the terminal branches of the ICA and caudally by the CCuA, which joined the BA. This closed circular pattern provides an efficient collateral network that enables a bidirectional flow between the carotid and basilar systems (2, 3). In contrast, ruminants exhibit a functionally modified circle due to the regression of the ICA and the presence of the carotid rete mirabile (27, 28). Therefore, the dominance of the ICA and arterial circle in equine may reflect a species-specific vascular organization, maintaining direct carotid flow similar to that described in carnivores and primates (7, 29).

### 4.3 Rostral cerebral artery

The RCA supplies the cerebral cortex, including the frontal and parietal lobes and portions of the temporal lobes. The origins of the MCA and RChA from the proximal segment of the RCA in this study aligned with previous descriptions in equine studies (2, 3). A distinct RCoA linking the two RCAs was consistently identified in all examined specimens. Moreover, supplementary anastomotic connections between the RCAs were present in approximately 60% of the brains, suggesting a notable degree of vascular redundancy within the equine forebrain. The cortical branches of the RCA, including the orbitofrontal, frontopolar, and frontal arteries, exhibited a branching configuration comparable to that documented by Böing et al. (3), who reported a hierarchical arrangement on the medial surface of the equine cerebral hemisphere.

### 4.4 Middle cerebral artery

The MCA is the largest branch of the carotid system and constitutes the primary artery of the lateral cerebral surface. The lateral bifurcation of the MCA into the rostral and caudal trunks corresponded with the configuration reported by Böing et al. (3) who described six principal cortical branches distributed across the dorsolateral cortex. In our specimens, the rostral trunk supplied the lateral and dorsal frontal lobes through the orbital, inferior, middle, and superior frontal arteries. The caudal trunk provided blood to the parietal and temporal regions via the rostral and caudal temporal branches and several parietal arteries. The presence of a shared parietal trunk and an accessory middle parietal artery in 40% of specimens indicated intraspecific variability, comparable to patterns observed in other mammals (7, 30). The lack of a duplicate root in the MCA, which is common in ruminants and camels (20, 21), highlights the structural stability of the equine MCA.

### 4.5 Caudal cerebral artery

The CCeA originated from the CCuA in all examined specimens, consistent with previous observations by Moraes et al. (2) and Hayah (11). This artery perfuses the occipital and caudal temporal lobes and serves as the principal source of blood to the posterior hemispheric zone. The consistent presence of the medial and lateral occipital branches concurs with classical accounts of the caudal cerebral distribution in large mammals (7). Variation in the origins of the medial rostral and medial caudal temporal arteries likely reflects individual variability in vascular organization rather than a consistent species-level pattern.

### 4.6 Arterial anastomoses

In our study, anastomosing connections were commonly observed between both RCAs. These associations, combined with the convergence of the CCuA and BA, create a robust collateral circuit that stabilizes cerebral blood flow even in the case of compromised partial arteries. The completeness of the circle of Willis and the dense anastomotic network constitute a functional system that protects against localized ischemia by rendering alternative flow from the contralateral or caudal channels (2, 7). In animals, such as ruminants, where the ICA is replaced by a rete mirabile, this redundancy occurs outside the cranial cavity and exhibits reduced responsiveness to changes in blood flow (5, 6, 27, 28).

Anastomotic connections within the circle of Willis and between the rostral cerebral arteries may contribute to collateral circulation and maintenance of cerebral perfusion under conditions of vascular compromise. These configurations may be relevant in ischemic or obstructive events, although their functional significance requires further investigation.

In equine clinical practice, such vascular arrangements may be particularly relevant in conditions such as guttural pouch mycosis, where rupture of the internal carotid artery can lead to severe hemorrhage. In these cases, retrograde flow through collateral pathways may contribute to contralateral vascular involvement (2). Various surgical and endovascular techniques, including unilateral and bilateral internal carotid artery occlusion, have been described for the management of guttural pouch mycosis, in which an understanding of collateral circulation is critical to preventing cerebral ischemia (22, 23).

The present study has several limitations that should be considered. First, the sample size (*n* = 10), although consistent with previous anatomical studies (2, 3), limits the generalizability of the findings. Second, the study is primarily descriptive and does not include quantitative or morphometric analyses, which could further characterize vascular variability. Third, the latex injection technique may not adequately fill small-caliber vessels, potentially limiting the visualization of finer arterial structures. Although contrast-enhanced CT and other imaging techniques may provide a more physiologic representation of vascular structures, they were not available for this study. Latex injection with dissection enables direct visualization of arterial branching patterns with high spatial resolution, although formaldehyde fixation may affect tissue properties. Advanced imaging approaches, including CT angiography and iodine or barium-based contrast methods, could complement these findings (12).

Future studies incorporating larger sample sizes, quantitative approaches, and advanced imaging modalities would further strengthen the interpretation of these findings. Accordingly, the present observations should be considered representative of the examined specimens rather than definitive of the species.

## 5 Conclusion

This study offers a detailed description of the arterial architecture supplying the equine brain. The rostral, middle, and caudal cerebral arteries arise from the circle of Willis and collectively ensure a balanced arterial supply to the entire cortical surface. These findings contribute to a more refined understanding of the neurovascular architecture in equines, providing a detailed anatomical reference that complements existing knowledge in equine neuroanatomy. Future investigations employing advanced imaging modalities and detailed morphometric assessments are required to elucidate the functional implications of these vascular configurations in the equine brain.

## Data Availability

The raw data supporting the conclusions of this article will be made available by the authors, without undue reservation.
